# Occupational Chemical Exposure and Breast Cancer Risk According to Hormone Receptor Status: A Systematic Review

**DOI:** 10.3390/cancers11121882

**Published:** 2019-11-27

**Authors:** Veruscka Leso, Maria Luigia Ercolano, Dante Luigi Cioffi, Ivo Iavicoli

**Affiliations:** Department of Public Health, Section of Occupational Medicine, University of Naples Federico II, Via S.Pansini 5, 80131 Naples, Italy; ercolanoluisa@gmail.com (M.L.E.); danteluigicioffi@hotmail.com (D.L.C.); ivo.iavicoli@unina.it (I.I.)

**Keywords:** breast cancer, hormone receptor status, estrogen receptor, progesterone receptor, occupational chemical exposure, workplace risk factors, risk management

## Abstract

Breast cancers include a heterogeneous group of diseases with clinical behaviors that may vary according to the hormonal receptor status. However, limited knowledge is available on the role of breast cancer environmental and occupational risk factors in the onset of specific molecular disease phenotypes. Therefore, the aim of this review was to provide an overview on the possible correlation between occupational chemical exposures and breast cancers with a specific receptor pattern. Pubmed, Scopus, and ISI Web of Science databases were systematically reviewed to identify all the studies addressing chemical exposure in workplaces and risk of breast cancer classified according to the presence of estrogen and/or progesterone receptors. Some positive associations were reported between solvent, polycyclic aromatic hydrocarbon, organophosphoric insecticide, and synthetic fiber exposure and estrogen receptor-positive cases, while other investigations demonstrated a relationship with receptor-negative tumors or failed to detect any significant effect. Overall, further investigation should overcome limitations due to the self-reported information on work histories, the chemical classification in general categories, and the lack of environmental or biological monitoring exposure data. This may support the development of suitable and individually “tailored” occupational risk assessment and management strategies to protect the health of exposed workers, particularly those with hypersusceptibility conditions.

## 1. Introduction

Breast cancer is the most frequently diagnosed female tumor and the leading cause of malignancy mortality in women [1]. About 2 million new cancer cases and 626,000 related deaths have been estimated worldwide in 2018 [2]. Therefore, breast cancer represents a global, public health priority. Breast tumors include a heterogeneous group of diseases characterized by different histological types and molecular features that may be responsible for highly variable clinical behavior [3]. In this latter regard, tumoral subtypes with diverse hormonal receptor status, i.e., the presence or the absence of estrogen (E) and progesterone (P) receptors, may vary for disease aggressiveness, response to therapy, prognosis, and possibility of relapse [4].

Scientific evidence supports the role of numerous lifestyle, genetic, physiological, and pharmaceutical risk factors in breast cancer pathogenesis, and some positive associations have also been found with environmental and occupational exposures [5]. Indeed, employment in different workplace sectors, like farming, plastic production, metal working, chemical and rubber manufacturing, and healthcare, has been reported to increase the risk of breast tumors. Different risk factors may be responsible for such increase. These may include job organizational aspects, i.e., shift or night shift works; physical agents, i.e., ionizing radiation; but also the exposure to chemical substances, as suggested by the increased risk of breast cancer reported in workers exposed to i.e., a variety of solvents, pesticides, and ethylene oxide [5,6,7,8].

However, the above-mentioned different tumoral subtypes, and their variable clinical outcomes, require defining whether breast cancer risk factors, including workplace chemical exposure, may be related to diverse disease phenotypes, particularly concerning hormonal receptor status [9]. From a public and occupational health perspective, this seems an even more important issue, considering the increased levels of chemical exposure occurring in general living and occupational environments and the endocrine disruptive potential of some compounds. i.e., bisphenols, phthalates, pesticide, insecticides, metals, and solvents, that may be involved in the proliferation of the hormonal dependent mammary epithelium and cancer onset [10,11]. In this context, the aim of this review was to provide a comprehensive overview on the association between certain exposures to chemical substances in occupational fields and the onset of breast tumors defined by a characteristic receptor pattern. This may be important to define suitable risk assessment and management strategies for the workplace, aimed to protect the health of workers and particularly of those women with known hypersusceptibility conditions that many need deeper occupational health attention.

## 2. Materials and Methods

**Figure 1:**
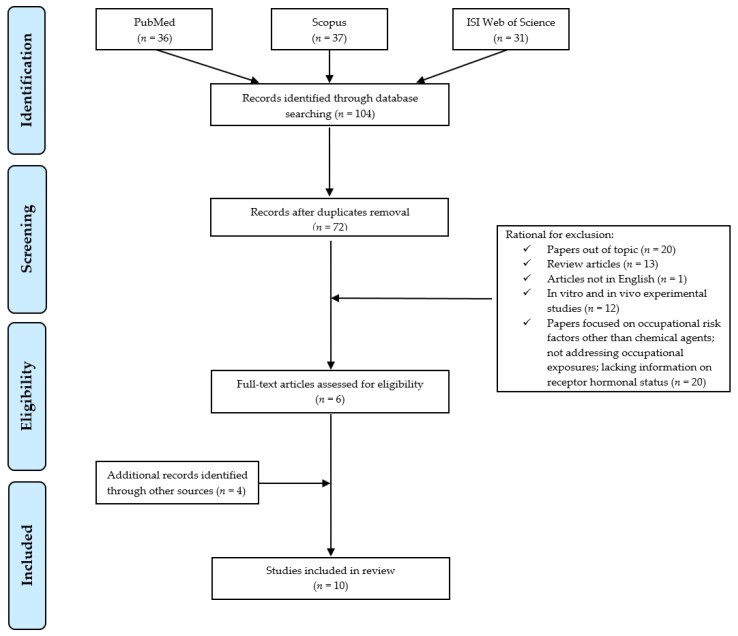
Flow diagram of literature search.

The preferred reporting items for systematic reviews and meta-analyses statement criteria (PRISMA) [12] were followed for the search and review processes (Figure 1). PubMed, Scopus, and ISI Web of Science databases were searched to identify occupational studies, published until 19 October 2019, addressing chemical exposures and risk of breast cancer according to hormone receptor status. Search terms (“occupation* OR “workplace”) AND “chemical*” were used to identify the context, “breast cancer”’ for the investigation outcome, AND (“hormone*” OR “receptor*”) AND (“estrogen” OR “progesterone”) to restrict the field to publications reporting data on cancer hormonal receptor status in their results.

Two of the authors independently examined all titles and abstracts retrieved and selected articles that met the inclusion criteria. These included all types of human peer-reviewed research articles (i.e., cross-sectional, cohort, case-control, or retrospective studies), published in English and exploring the relationship between workplace chemical exposure and breast cancer with specific regard to hormone receptor status. Exclusion criteria were applied for in vitro and in vivo experimental studies, reviews, articles published in languages other than English, studies on the risk of breast cancer in relation to occupational risk factors other than chemicals, not focused on workplace settings, or lacking hormone receptor information. The preliminary search retrieved 36 references through PubMed, 37 results through Scopus, and 31 results through ISI web of Science, for a total of 104 articles. After removal of duplicates, 72 articles remained. Among those, studies that did not meet the inclusion criteria were excluded according to the following reasons: 20/72 were removed because studies out of the topic from the title and abstract analysis; 13/72 were excluded as review articles, 1/72 was eliminated as not in English, 12/72 as experimental studies on in vitro or in vivo models, and 20/72 were not included as studies on breast cancer focusing on other occupational risk factors, or not related to occupational setting or lacking receptors data. Indeed, 6 publications could be identified in this preliminary phase. The manual assessment of the reference list accompanying published articles was employed to further supplement the citation pool of relevant publications identified in the literature and allowed to include 4 additional eligible articles. Overall, a total of 10 publications were suitable for review.

## 3. Results

Different studies explored the relationship between occupational chemical exposures, including solvents [13,14,15,16,17], polycyclic aromatic hydrocarbons (PAH) [14,18], insecticides [19,20], synthetic fibers [14], aromatic and heterocyclic amines (AHA) [21], or a miscellaneous of substances [22], and breast cancer risk with specific attention to different hormonal receptor subtypes (Table 1). Although conflicting results emerged from our review, some interesting information could be pointed out as summarized in the following paragraphs.

### 3.1. Occupational Exposure to Solvents

Organic solvents are lipophilic chemicals that can easily accumulate within the fat tissue of the breast and may play a role in breast carcinogenesis [23]. A previous study performed by Petralia et al. [13] analyzed the risk of premenopausal breast cancer in women occupationally exposed to benzene with or without associated PAH exposure, in comparison to referents never exposed to these substances. The risk of the disease resulted significantly associated with “ever” being exposed to such chemicals. When results were stratified by ER status, a significantly increased risk was found for ER-positive cases in all exposed groups. When the characteristics of the exposure to benzene, with possible PAH co-exposure, were analyzed, the average probability (low or medium to high), duration (<4 or ≥4 years) and cumulative levels (low or medium to high) resulted able to significantly increase the risk for ER-positive cancers [13]. No apparent differences in risk between ER-positive and ER-negative breast tumor cases could be detected when benzene exposure alone was considered, suggesting that possible interactions between chemicals may affect results. However, the limited number of cases and the lack of direct measures of exposures in this study require caution for a correct interpretation of the results.

Labreche et al. [14], in a case-control study conducted in Canada between 1996 and 1997, found no significant alterations in the overall post-menopausal cancer risk induced by lifetime or early (<36 years of age) exposure to organic solvents. Conversely, when the ER and/or PR status was assessed, a significantly increased risk was reported for ER-positive/PR-negative tumors following lifetime exposure to organic solvents with reactive metabolites, and particularly when it occurred before 36 years of age. Interestingly, as duration of exposure was considered, the risk for such type of cancers tripled for each 10-year increase in duration of exposure to organic solvents [14].

The risk of breast cancer in relation to occupational exposure to organic solvents and benzene alone, was investigated in cancer cases diagnosed between 2000 and 2003 in Poland and compared to controls who participated in a large population-based investigation [15]. A weak, marginally significant increase in breast cancer risk could be only determined in organic solvent-exposed women compared to unexposed subjects. No significant trend with increasing levels of exposures, assessed as the likely quantity of organic solvents used or as the estimated benzene concentrations in air, was found. When the tumor receptor subtype was considered, organic solvent exposure resulted to be significantly associated with ER-negative/PR-negative cases, but not with ER-positive/PR-positive ones. This may suggest a mechanism other than hormonal for tumor promotion. However, no exposure–response gradient could be detected for the association observed.

Interestingly, when the relationship between occupational exposure to solvents and breast cancer risk was prospectively analyzed in a large cohort of women who had a family history for the tumor, no significant association could be detected [16]. Results by ER status revealed an overall, non-significant changes in ER-positive tumors, with respect to either frequency or duration of solvent exposure. Significant increase in ER-positive cancer was demonstrated among women whose first solvent job was before 1980 and also among those exposed to solvents before their first birth compared to women who started working in solvent jobs after their first birth, exposed nulliparous, or unexposed controls. Concerning the relationship between job tasks and breast cancer development, clinical laboratory technologists and technicians who worked with solvents showed an increased risk for ER-positive cancers, while non-significant changes were observed for solvent-exposed maids and housekeeping cleaners as well as for solvent exposed women in productive occupations [16].

In a population-based case-control study, Glass et al. [17] compared 1205 women diagnosed with breast cancer between 2009 and 2011 with 1789 controls and assessed their exposure to solvents to verify possible relationship with cancer development. They found a non-significant increase in the risk of breast cancer among women professionally exposed to aliphatic and aromatic solvents, although there was no significant relationship with hormonal receptor status.

### 3.2. Occupational Exposure to Polycyclic Aromatic Hydrocarbons

Polycyclic aromatic hydrocarbons are by-products of combustion involving organic matter that can accumulate in mammary tissues, potentially contributing to cancer risk [24]. Petralia et al. [13] compared the risk of premenopausal breast cancer in women occupationally exposed to PAHs with or without benzene, with the risk in unexposed referents. The exclusive PAH exposure was not associated with an increased risk of breast cancer, which was conversely detected for co-exposure with benzene. When results were stratified by ER status, non-significant changes were found for women exclusively exposed to PAH, while significant increase for ER-positive tumors could be detected in cases of co-exposure.

Labreche et al. [14] failed to detect significant associations between PAH exposure and the overall postmenopausal breast cancer risk. However, PAHs from petroleum resulted significantly associated with elevated risks for ER-positive/PR-negative tumors for both lifetime and earlier (<36 years) exposure. Conversely, a more recent population-based case-control study, conducted in Canada, demonstrated that exposure to any levels of PAH was associated with an overall increased risk of breast cancer, in comparison to never exposed women. However, no difference in breast cancer risk was observed when tumors were stratified by hormone receptor status [18].

### 3.3. Occupational Pesticide Exposure

Women engaged in agricultural work may experience exposure to several insecticides, both directly (i.e., mixing, applying) and indirectly (from working in fields containing pesticides residues) [25]. Lerro et al. [19] evaluated personal use of organophosphate insecticide (OPs) and breast cancer incidence among 30,003 spouses of pesticide applicators in the US Agricultural Health Study. In this study, use of “any OP” was significantly associated with breast cancer, while regarding specific OPs, only chlorpyrifos and terbufos were associated with non-significantly elevated risk of breast cancer. Concerning hormonal receptor status, chlorpyrifos was associated with a significantly increased risk of ER-negative/PR-negative breast cancer [19]. In an update of the previous study on 30,594 wives of pesticide applicators, primarily farmers, Engel et al. [20] demonstrated that ever personal use of any insecticide, including carbamates, organochlorines, and OPs, was not associated with risk of breast cancer. However, focusing on specific substances, a significant association was detected only for chlorpyrifos and terbufos as previously reported. When the analysis was stratified according to the ER/PR status, no significant differences could be found.

### 3.4. Other Chemical Exposures

Exposures to synthetic fibers, in particular acrylic, rayon and nylon fibers, and wool fibers were not significantly associated with postmenopausal breast cancer as a whole [14]. However, analyzing hormonal receptor status, lifetime exposure to acrylic and rayon fibers increased the risk for ER-positive/PR-negative cancers, while early exposure (<36 years age) to acrylic fibers increased the risk for both ER-positive/PR-positive and ER-positive/PR-negative phenotypes. Nylon and synthetic fiber exposure at age <36 years was associated with an increased risk for tumors positive for both receptors, while rayon fibers significantly increased negative status.

A population-based case-control study in Germany assessed exposure to aromatic and heterocyclic amines (AHA) from self-reported work with rubber, hair dyes, leather, textiles, paper, painting, and tar. No significant effects were found for occupational AHA exposure on cancer risk both when overall breast cancer cases, as well as E and P positive/negative receptor tumors were assessed [21]. When women occupationally exposed to different types of chemicals were prospectively analyzed for risk of tumor development [22], only a borderline excess risk of invasive breast cancer in women ever exposed to dyes or inks was evident. Exposure to soldering materials was associated with a significantly increased risk of premenopausal breast cancer. However, no significant differences could be detected for hormone receptor status. A borderline increased risk of hormone receptor-positive breast cancer was only observed for women in the highest quartile of exposure to paints [22].

## 4. Discussion

This review represents a first attempt to provide a comprehensive overview on the possible association between occupational chemical exposure and the risk of developing breast cancer with particular hormone receptor status. This seems to be a challenging public and occupational health issue, considering the high prevalence of breast cancers in the female population and the increasing levels of chemical exposure occurring in both general living and workplaces. Unfortunately, only a limited number of studies investigated breast cancer risk associated with chemicals in workplaces with a specific focus on tumor receptor subtypes. This aspect seems to be an important issue to be explored, since some studies failed to detect a significant increase in the “overall” risk of breast cancer with respect to chemical exposure, while could demonstrate significant changes in specific tumor ER, PR positive or negative neoplasms [14,16].

In this regard, conflicting evidence emerged. In fact, some positive associations have been reported between solvent, PAH, OP insecticide, and synthetic fiber exposure and ER-positive [13,14,16], as well as ER-positive/PR-negative tumors [14]. Other investigations demonstrated possible relationships between solvents and OP insecticides and ER-negative/PR-negative cases [15,19], or failed to detect any significant association [17,18,20,21,22].

However, some critical issues should be considered to cautiously interpret the obtained results. Generally, most of the studies derived exposure information from self-reported work histories, job titles, or job-exposure matrices. This may characterize a misclassification bias in assessing exposure–disease relationship, since self-reported information may overestimate exposure, but may also fail to identify all conditions potentially occurring in workplaces. None of the revised studies provided a reliable environmental or biological monitoring assessment of the exposure, thus preventing the definition of a suitable dose-response relationship.

Furthermore, in some cases, only the overall general class of chemicals was adopted as a “proxy” to assess exposure, i.e., organic solvents and not individual components [14,15,16]. This makes it difficult to assess the weight of individual chemical agents in determining the risk of breast cancer, as suggested by the differences reported in tumor subtypes following i.e., benzene, PAH alone, or in combination [13]. This aspect concerns not only chemical co-exposures, but also exposure to a number of occupational risk factors, i.e., shift or night shift work, ionizing radiations, known for their influence on breast cancer development [8,26,27]. Furthermore, the existence of several non-professional risk factors for the onset of breast cancer (e.g., family history, reproductive and hormonal factors, lifestyle, environmental factors) makes it even more challenging to define the contribution of occupational chemical exposure in disease development. In this regard, a couple of prospective studies analyzed the association between ER-positive tumors and chemical exposure in women with a family history of breast cancer [16,22], reporting a significant increase only for those exposed to solvents before 1980 (periods with probable greater levels of exposure) and before their first birth. These findings may support the possible different impact that occupational chemical agents may have according to the stages of life during which exposure occurs. Women in premenopausal age, in which breast cells are still proliferating, could be more sensitive to the adverse effects of chemicals compared to women in postmenopausal period. As a confirmation, solvent exposure in early life, <36 years, induced a more evident increase of ER-positive/PR-negative tumors with respect to lifetime exposure [14], and only solvent exposure occurring before and in combination with the pregnancy proliferative changes demonstrated an influencing role in the development of ER-positive breast cancer types [16]. Moreover, acrylic fibers were reported to increase the risk of different cancer hormone subtypes according to the lifetime or earlier exposure in life (<36 years) [14]. It should be also considered that specific environmental chemical exposures may have a different weight in the development of breast cancer depending on the specific windows of susceptibility through an overall woman’s life course i.e., the prenatal or pregnancy period as well as the menopausal transition time [28]. These aspects should be better investigated to verify the existence of a different carcinogenic susceptibility of the breast tissue in relation to exposure before or after full differentiation, in order to adopt preventive measures focused on specific groups of women and particular types of tumors.

Ekenga et al. [16] also identified clinical laboratory technologists and technicians as occupations with elevated ER-positive breast cancer risk associated with solvent exposure. This may be related to the high levels of exposure in different activities, like applying topical cleansers and antiseptics, sterilizing instruments, but also to the inadequate adoption of collective and personal protective measures, that should be carefully evaluated when assessing exposure in occupational settings.

Various mechanisms of carcinogenicity have been proposed for chemical exposure, including the possibility for chemicals, like solvents, to be converted by breast cells into reactive oxygen species, involving free radicals and epoxides, and exert local direct effects leading to cancer induction [14]. Concerning particular types of breast cancer subtypes, the increases found with exposures to i.e., organic solvent, PAHs, and synthetic fibers among ER-positive/PR-positive tumors support the idea that certain occupational chemicals present in the workplace, acting as endocrine disruptors, in particular xenoestrogens, can favor the onset of hormone receptor-positive breast cancers [29]. Moreover, it cannot be excluded that chemical exposure may induce epigenetic alterations characterized by changes in DNA methylation, histone modifications, and microRNA expression, which may play a role in sustaining cancer hallmarks [30]. From public and occupational health perspectives, the possible impact that chemicals may have on biological pathways leading to cancer risk increase needs deep investigation as exposure to chemicals has become globally pervasive.

## 5. Conclusions

Although this review pointed out interesting results, the relationship between workplace chemical exposure and risk of breast cancers with specific hormone receptor status need further confirmation. Environmental and biological monitoring exposure assessment should be performed to define an adequate dose–response relationship. Additionally, exposure metrics including duration, years of first exposure, and periods of life characterized by a greater susceptibility to the development of specific cancer types, should be deeply defined. Moreover, future research should be aimed to elucidate the complex interplay, between individual non-modifiable, and modifiable risk factors, i.e., lifestyle factors, as well as environmental and occupational features, that could lead to the development of neoplasms with a particular hormonal receptor status. Overall, this may guide the development of suitable and individually “tailored” occupational risk assessment and management strategies aimed to protect the health of workers, particularly those with increased risk due to lifestyle, reproductive, and genetic profiles.

## Figures and Tables

**Table 1:** Studies addressing exposures to chemicals and risk of breast cancer according to hormone receptor status.

References	Country (Period of Investigation)	Occupational Chemical Exposure; Job Tasks	Investigated Population	Methods	Results
Petralia et al. [13]	USA (1986–1991)	Benzene and PAHs PAH exposure (no benzene): traffic, shipping and receiving clerks, inspectors, testers, graders in motor vehicles industry Benzene exposure (no PAH): laboratory technologists and technicians, painters, sculptors, artists, assemblers in motor vehicle industry PAH and benzene: bus truck and stationary engine mechanics, molding and casting machine operators, garage, and service station occupation	Women with premenopausal breast cancer (age: ≥40 years) (n.301) Referents matched by age and country of residence (n.316)	✓ Tumors with ER >10 fmol/mg or cells with ER > 10% classified as ER-positive. ✓ Personal, medical, and occupational histories obtained by interviews. ✓ Exposure assessed by job-exposure matrices	✓ Overall breast cancer risk increased with ever being exposed to chemicals. Benzene (possible PAH co-exposure) (OR: 1.91; 95%CI 1.18–3.08); PAH (possible benzene co-exposure) (OR: 1.82; 95%CI 1.02–3.16); PAH and benzene -all exposed groups (OR: 2.01; 95%CI 1.08–3.75); exclusively benzene (OR: 1.70; 95%CI 1.17–2.92) ✓ No increased overall breast cancer risk following exposure to PAH alone (OR: 1.01; 95%CI 0.55–3.45) ✓ Increased risk of ER-positive cases in all exposure, except benzene alone: PAH (possible benzene co-exposure) (OR: 2.27; 95%CI 1.14–4.54); Benzene (possible PAH co-exposure) (OR: 2.29; 95%CI 1.27–4.13), PAH and benzene (OR: 2.54; 95%CI 1.28–5.04); Exclusively benzene (OR: 1.74; 95%CI 0.72–4.21). ✓ No increased risk for ER-positive tumors for PAH exposure alone (OR: 0.78; 95%CI 0.12–5.03) ✓ ER-negative cases: PAH (possible benzene co-exposure) (OR: 1.12; 95%CI 0.47–2.64); Benzene (possible PAH co-exposure) (OR: 1.49; 95%CI 0.74–3.02), exclusively PAH (OR: 1.79; 0.46–8.52); PAH and benzene (OR: 1.28; 95%CI 0.52–3.15); Exclusively benzene (OR: 2.10; 95%CI 0.87–5.53).
Labreche et al. [14]	Canada (1996–1997)	Organic solvents in broad categories Organic solvents: gasoline, diesel, jet fuel engine emissions and mineral spirits Monoaromatic hydrocarbons: benzene, toluene, xylene, and styrene Compounds containing PAH from petroleum: carbon black, petroleum soot, jet fuel engine emissions, oil-based cutting fluids and more Other chemicals: wool, acrylic and rayon fibers	Women with postmenopausal primary breast (n.556; 50–75 years old) Women with other cancers matched by age (n.613)	✓ Personal, medical, and occupational histories obtained by interviews. ✓ Exposure assessed translating all jobs into a set of exposure indices by a team of experienced industrial hygienists and chemists ✓ Hormonal receptor status categorized as positive or negative for ER and/or PR, as provided in the pathology reports.	✓ No significant alterations in risk of postmenopausal breast cancer with lifetime (OR: 1.14; 95%CI 0.80–1.62), or early (<36 years) exposure to organic solvents (OR: 1.30; 95%CI 0.65–2.60). ✓ Organic solvents increased the risk for ER-positive/PR-negative tumors in early exposures (<36 years) (OR: 3.31; 95%CI 1.07–10.20) ✓ PAHs from petroleum associated with increased risk of ER-positive/PR-positive tumors for both lifetime (OR: 1.65; 95%CI 0.97–2.83) and early (OR: 3.00; 95%CI 1.10–8.13) exposure. ✓ Acrylic fibers: For ER-positive/PR-negative tumors the risk tripled for each 10-year increase in duration on exposure to organic solvents and more than doubled for each 10-year increase in duration of exposure to monoaromatic hydrocarbons. Exposure to PAHs from petroleum tripled the risk for ER-positive/PR-positive tumors.
Peplonska et al. [15]	Poland (2000–2003)	Organic solvents and benzene “Organic solvents” include aromatic, aliphatic, chlorinated hydrocarbons, ketones, organic acid esters, petroleum distillates.	Female newly diagnosed with in situ or invasive breast cancer (n.2383; age: 20–74 years) Controls matched to the cases by city of residence and age (n.2502).	✓ Personal, medical, and occupational histories obtained by questionnaires, medical records, and pathology forms. ✓ Occupational exposure scored as intensity, probability, and duration. ✓ ER and PR status determined by immunohistochemistry or biochemical methods.	✓ Non-significant increase in breast cancer risk in women ever exposed to organic solvents (OR: 1.16; 95%CI 0.99–1.4). No significant changes for benzene exposure alone (OR: 1.00; 95%CI 0.8–1.3) ✓ None of the exposure metrics showed evidence for a exposure-response risk of breast cancer. ✓ Exposure to organic solvents significantly associated with an increased risk for negative ER and PR tumors (OR 1.40; 95%CI 1.1–1.8).
Ekenga et al. [16]	USA and Puerto Rico (2003–2009)	Solvent exposed occupations Building and grounds cleaning and maintenance; education, training, and library; food preparation and serving related; healthcare practitioner and technical; management; office administrative and support; production occupations.	Women enrolled in the Sister Study (n. 47,661 sisters of women with breast cancer) occupationally exposed to solvents Women diagnosed with breast cancer during follow-up (n. 1798)	✓ Personal, medical, and occupational histories obtained by interviews and questionnaires. ✓ Most commonly reported solvent-exposed occupations classified into major categories. ✓ Data on receptor status obtained by medical record, pathology report data or self-reported.	✓ No increased risk of invasive breast cancer among women occupationally exposed to solvents (HR: 1.04; 95%CI 0.88–1.24). ✓ Non-significant increase for ER-positive tumors in exposed women (HR: 1.15; 95%CI 0.95–1.39) ✓ ER-positive tumors significantly associated with exposure before the birth of their first child (HR 1.39; 95% CI 1.03–1.86) and first exposure to solvents occurred before 1980 (HR: 1.28; 95%CI 1.01–1.62). ✓ Significantly elevated risk for ER-positive cancers associated in clinical laboratory technologists and technicians who worked with solvents (HR: 2.00, 95%CI: 1.07–3.73)
Glass et al. [17]	Australia (2009–2011)	Solvents Benzene, other aromatic, aliphatic, chlorinated solvents, and alcohol	Women with first incident invasive primary breast cancer (n.1205; aged 18–80 years) Randomly selected age-matched controls (n.1789)	✓ Personal, medical, and occupational histories obtained by interviews and questionnaires. ✓ Occupational solvents exposure assessed using telephone interview and the web-based application OccIDEAS. ✓ Information on ER status obtained by interviews and questionnaires.	✓ Non-significant increase in the risk of breast cancer among women professionally exposed to aliphatic (OR 1.21; 95% CI 0.99-1.48) and aromatic (OR 1.21; 95% CI 0.97–1.52) solvents ✓ No differences detected for hormonal receptor status.
Lee et al. [18]	Canada (2005–2010)	Polycyclic aromatic hydrocarbons Food-service industry (>20%)	Women aged 40–80 years diagnosed with in situ or invasive breast cancer (n.1130). Women recruited from the Breast Screening Programme as age-matched controls (n.1169)	✓ Personal, medical, and occupational histories obtained with questionnaires ✓ Exposure to PAHs assessed by a job exposure matrix based on a statistical model	✓ Exposure to any level of PAHs associated with a significantly increased risk of breast cancer (OR: 1.32, 95%CI 1.10–1.59) ✓ Evidence of increased risk with duration of exposure apparent for medium or high exposure levels (the longest duration: OR: 1.41, 95%CI: 1.10–1.81) and high exposure levels (the longest duration: OR: 1.45, 95%CI: 1.10–1.91). ✓ No difference in breast cancer risk observed by receptor status
Lerro et al. [19]	USA (Enrollment: 1993–1997; Follow-up: until 2010–2011)	Organophosphate (OP) insecticides OP insecticides: chlorpyrifos, coumaphos, diazinon, dichlorvos, fonofos, malathion, parathion, phorate, terbufos, trichlorfon	Spouses of private pesticide applicators (n. 30,003) Women with diagnosed breast cancer during the follow up period (n. 718)	✓ Personal, medical, and occupational histories obtained by questionnaires. ✓ Pesticides exposure assessed by questionnaires ✓ Incident breast cancer cases ascertained through population-based cancer registries.	✓ Any OP use associated with an elevated risk of breast cancer (RR: 1.20, 95%CI 1.01–1.43) ✓ Chlorpyrifos use (RR: 1.41, 95% CI: 1.00–1.99) and terbufos use (RR: 1.52; 95% CI 0.97–2.36) associated with non-significantly elevated risk of breast cancer ✓ Among post-menopausal women, significantly elevated risk of breast cancer associated with use of any OP (RR: 1.27, 95%CI 1.00–1.62), and non-significantly elevated breast cancer risk associated with chlorpyrifos (RR: 1.53, 95%CI 0.96–2.44) and terbufos (RR: 1.73; 95%CI 0.93–3.21). ✓ Chlorpyrifos was associated with a significantly increased risk of ER-negative/PR-negative breast cancer (RR: 2.26, 95% CI: 1.07–4.75)
Engel et al. [20]	USA (Enrollment: 1993–1997; Follow-up: until 2010–2011)	OP insecticides	Spouses of private pesticide applicators (n.30594) Women with diagnosed incident breast cancer during follow-up period (n.1081)	✓ Personal, medical, and occupational histories obtained by questionnaires and interviews. ✓ Pesticides exposure assessed by questionnaires ✓ Incident breast cancer cases ascertained through population-based cancer registries.	✓ Ever personal use of any insecticide associated with risk of breast cancer (HR: 1.0; 95%CI 0.7–2.9) ✓ Significant association between breast cancer risk and ever use of chlorpyrifos (HR: 1.4, 95%CI: 1.0–2.0) and terbufos (HR: 1.5, 95% CI: 1.0–2.1). ✓ No significant differences in risk related to the woman’s use of insecticides according to ER tumoral status.
Rabstein et al. [21]	Germany (2000–2004)	Aromatic and heterocyclic amines (AHA) Job activities: developing of films, rubber industry, using dyes, painting, working with tar.	Incident breast cancer cases from the GENICA study, a German population-based case-control study (n.1155) Age-matched controls (n.1143)	✓ Data on breast cancer risk factors obtained with interviews. ✓ Expert rating applied to assess possible occupational exposure based on self-assessed tasks. ✓ Immunohistochemical staining of breast-cancer tissues: ER+ and PR+ when ≥10% cells showed nuclear staining.	✓ No significant association for occupational AHA exposure and risk of overall breast cancer (OR: 1.05; 95%CI 0.70–1.56 for >1-year exposure vs. none or < 1 year). ✓ No significant association for occupational AHA exposure and risk of ER-positive (OR: 1.26; 95%CI 0.81–1.95 for >1 year exposure vs. none or < 1 year) or ER-negative cancers (OR: 0.81; 95%CI 0.36–1.82 for >1 year exposure vs. none or < 1 year) ✓ No significant association for occupational AHA exposure and risk of PR-positive (OR: 1.24; 95%CI 0.79–1.94 for >1 year exposure vs. none or < 1 year) or PR-negative cancers (OR: 0.82; 95%CI 0.38–1.76 for >1 year exposure vs. none or < 1 year)
Ekenga et al. [22]	USA and Puerto Rico (2003–2009)	Different chemical substances Acids, dyes or inks, gasoline or other petroleum products, glues, or adhesives, lubricating oils, metals, paints, pesticides, soldering materials, solvents and stains or varnishes.	Women enrolled in the Sister Study (n. 45,674 sisters of women with breast cancer) occupationally exposed to different chemicals Women diagnosed with breast cancer during follow-up (n. 1966)	✓ Personal, medical, and occupational histories obtained by interviews and questionnaires. ✓ Cumulative exposure to each agent estimated as a function of frequency and duration of use; quartile cut points were used to assign participants to exposure categories ✓ Data on receptor status obtained by medical record, pathology report data, or self-reported.	✓ No significant associations between ever use of chemical agents and breast cancer risk. ✓ Significant association between occupational exposure to soldering materials and premenopausal breast cancer (HR: 1.8, 95%CI = 1.1–3.0). ✓ Women with cumulative exposure to gasoline or petroleum products ≥ the highest quartile cutoff had an elevated risk of total (HR: 2.3, 95% CI: 1.1–4.9) and invasive (HR: 2.5, 95% CI: 1.1–5.9) breast cancer compared with women in the lowest quartile. ✓ Risk estimates did not differ significantly by hormone receptor status. ✓ Exposure to gasoline or other petroleum products in the highest quartile associated with a non-significant increased risk of hormone-receptor positive breast cancer (Q4 vs. never used: HR: 1.4, 95% CI: 0.9–2.3; Q4 vs. Q1: HR: 2.5, 95% CI: 1.0–5.8). ✓ Borderline increased risk of hormone receptor-positive cancer observed for women in the highest quartile of exposure to paints (Q4 vs. never used: HR: 1.4, 95% CI: 1.0–2.0).
